# MicroRNAs as Prognostic Markers in Acute Coronary Syndrome Patients—A Systematic Review

**DOI:** 10.3390/cells8121572

**Published:** 2019-12-04

**Authors:** Jennifer Y. Barraclough, Michelyn Joan, Mugdha V. Joglekar, Anandwardhan A. Hardikar, Sanjay Patel

**Affiliations:** 1 Department of Cardiology, Royal Prince Alfred Hospital, 2050 Sydney, Australia; 2 Sydney Medical School, The University of Sydney, 2050 Sydney, Australia; 3 Heart Research Institute, 2042 Sydney, Australia; 4 NHMRC Clinical Trials Centre, Faculty of Medicine and Health, The University of Sydney, 2050 Sydney, Australia; mugdha.joglekar@ctc.usyd.edu.au (M.V.J.); anand.hardikar@ctc.usyd.edu.au (A.A.H.)

**Keywords:** miRNA, biomarker, prognosis, acute coronary syndrome

## Abstract

Background: The potential utility of microRNAs (miRNAs) in the diagnosis, prognosis, and treatment of multiple disease states has been an area of great interest since their discovery. In patients with cardiovascular disease, there is a large pool of literature amassed from the last decade assessing their diagnostic and prognostic potential. This systematic review sought to determine whether existing literature supports the use of miRNAs as prognostic markers after an Acute Coronary Syndrome (ACS) presentation. Methods: A systematic review of published articles from 2005–2019 using MEDLINE and EMBASE databases was undertaken independently by two reviewers. Studies addressing prognosis in an ACS population yielded 32 studies and 2 systematic reviews. Results/conclusion: 23 prospective studies reported significant differences in miRNA levels and 16 compared the predictive power of miRNAs. The most common miRNAs assessed included miR-133a, -208b, -21, -1, -34a, -150, and -423, shown to be involved in cell differentiation, apoptosis, and angiogenesis. Barriers to the use of miRNAs as prognostic markers include bias in miRNA selection, small sample size, variable normalization of data, and adjustment for confounders. Therefore, findings from this systematic review do not support the use of miRNAs for prognostication post-ACS beyond traditional cardiovascular risk factors, existing risk scores, and stratifications tools.

## 1. Introduction

Coronary artery disease (CAD) is the leading cause of mortality worldwide [1]. Advancements in early diagnosis and prognostication would allow for better risk stratification in an aging global population. Since the discovery of miRNAs, there has been great interest in their role in various disease processes, including atherosclerosis. As noncoding RNAs regulate posttranscription gene expression, the potential of miRNAs as a biomarker or as a mediator of the disease process has been identified [2]. This has yielded a multitude of studies in miRNA level characterisation and its various potential applications including diagnosis [3] and prognosis [4,5,6] in CAD.

Despite the large volume of existing literature on miRNAs in atherosclerosis, the vision of miRNA panels being used in clinical practice to quickly and accurately diagnose CAD has not become a reality. The availability of high-sensitivity cardiac Troponin T(hs-cTnT) allowing for early diagnosis of myocardial infarction (MI) makes it increasingly difficult for potential or novel biomarkers to compete with those utilized in current practice [7]. Furthermore, contradictory results, variations in methodology, and an abundance of underpowered studies have not facilitated inclusion of miRNAs in current diagnostic and prognostic algorithms [8]. Amongst these limitations as well as technological constraints, current large cohort studies, reviews [3], or meta-analyses [8] have similarly been unable to demonstrate the value of miRNAs in a clinical diagnostic setting over traditional biomarkers, although there is some suggestion they may be additive in diagnosis [9].

Nonetheless, cancer research has witnessed significant discoveries in identifying miRNAs as potential cancer biomarkers or treatment targets. Thus, interest in identifying miRNAs through discovery and validation of candidates that have diagnostic/prognostic potential is increasingly under investigation in patients presenting with Acute Coronary Syndrome (ACS). As such, this review discusses the current literature and outlines the need for further studies, with a particular focus on identifying miRNAs post-ACS as prognostic biomarkers alongside traditional cardiovascular risk factors, existing risk scores, and stratifications tools. 

## 2. Materials and Methods

**Figure 1:**
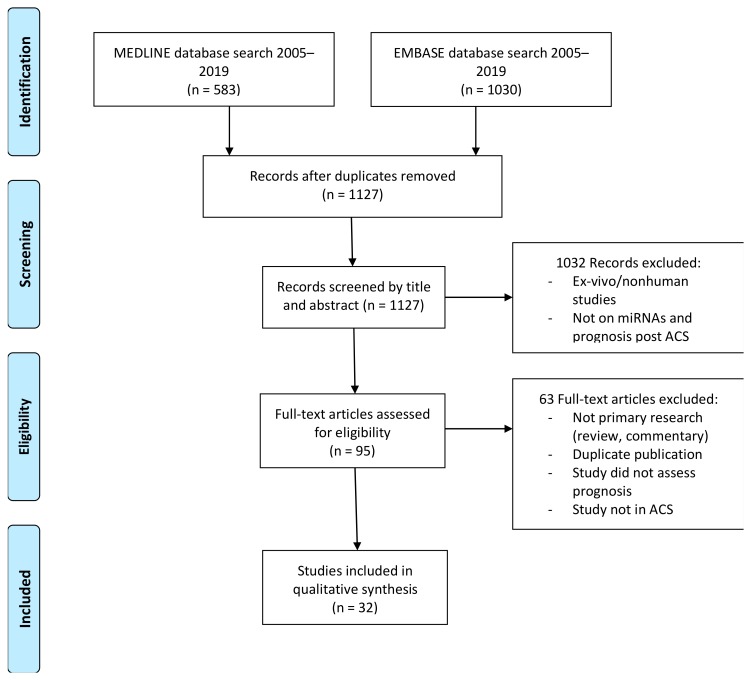
Flow diagram: prognostic utility of miRNAs in acute coronary syndrome—systematic review [10]. ACS: acute coronary syndrome.

Electronic databases accessible via the University of Sydney: MEDLINE and EMBASE via Ovid were used and supplemented by screening references of literature (via PubMed) deemed relevant to the current study. The strategy of the literature search is summarised in Figure 1.

A comprehensive search of the accessible published literature was conducted by combining key search terms using the Boolean term AND and search term variations of microRNAs, coronary artery disease/cardiovascular disease, and outcomes including morbidity/mortality using the Boolean term OR as keywords to maximize sensitivity with the last search taking place on 27 August 2019. The relevant MeSH (medical subject headings) terms or similar, available on MEDLINE and EMBASE, were used to further cast a wider net and to maximise sensitivity of our search for relevant pieces of literature. All records published up until August 2019 were included in our search, where the records were either in English or an English translation was available. 

However as only published studies in the public domain were accessed, this review is potentially biased in favour of positive findings which are more often published and skewed towards findings of significance for Caucasian populations given that most studies found involved predominantly Caucasian patients. This review was undertaken by two reviewers J.B. and M.J., who conducted the literature search and performed the data extraction of study characteristics and results (Table S1), which could be a source of bias despite data extraction being conducted at 2 separate time points and compared to minimise errors. Studies identified to be within the scope of the review were assessed for bias within studies. Risk of bias was undertaken by 2 reviewers J.B and M.J. The QUIPS (quality in prognosis studies) tool [11] was used to assess the quality of studies. The six main domains included study participation, attrition, prognostic factor, outcome factor, confounding and statistical analysis/reporting for determining the overall bias within each study and, thus, the value in this review. Data was also collected to assess bias across studies on miRNA selection (Table S1). 

There were initially 1127 articles identified (excluding ~630 duplicates automatically detected on EndNote). Due to the choice of maximising sensitivity over specificity in the initial electronic search, it then was necessary to determine inclusion and exclusion of literature by direct inspection of the titles and abstracts to assess for relevance to the aims of this study. 

Manual screening of the titles and abstract followed by full-text evaluation of the literature yielded 32 primary research articles/records (Figure 1) suitable for discussion within this review and two meta-analysis. Articles were evaluated for relevance to the research question, where studies not addressing the prognostic potential of miRNAs in the context of acute coronary syndromes, in vitro studies, and those containing nonhuman populations were excluded. Details such as the population, methods, findings, and limitations of the remaining records were catalogued (Table S1) for further discussion. Authors followed the PRISMA guidelines for systematic review.

## 3. Results and Discussion

**Figure 2:**
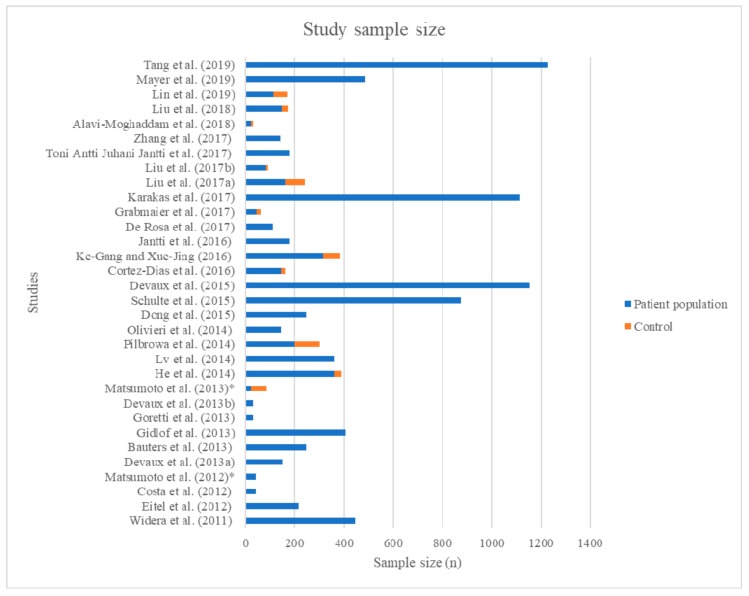
Populations of original research studies in miRNA as biomarkers for prognosis in acute coronary syndromes: Bar chart of original research articles included in this review, displaying patient population in blue and control population in orange.

As briefly mentioned above, 32 primary research records were found to be relevant to the research question (Table S1), and 2 recent systematic reviews with meta-analyses were found. Out of the studies, 29 were prospective and 3 were retrospective analyses (Figure 2) of patient populations. None of the three retrospective studies were included in the reviews. Study sizes ranged from 30–1199 persons, and follow-up times for the prospective populations ranged from 30 days to up to 6 years. Only 11 out of the 29 prospective studies deliberately recruited a healthy control population, only three of which recruited matched controls [12,13,14]. As we discuss, it is important to note the study limitations as outlined and detailed in Table S1 and factors including population size. 

In general, studies investigated miRNA levels in serum or plasma, sampled as close to diagnosis and treatment (percutaneous coronary intervention (PCI)) as relevant or up to 1 month after recruitment except for one study measuring miRNA 6–36 months post ACS [15] and quantified using Real-Time Polymerase Chain Reaction (RT-PCR). Outcomes analysed included but were not limited to all causes and cardiac mortality, subsequent myocardial infarction, angina, heart failure, left-ventricular ejection fraction, or contractility as a measure of function, arrhythmia, and hospitalisations. 

Bias within studies was assessed using the QUIPS tool. Out of the 32 studies, 11 had low risk of bias across the six domains assessed. High risk of bias was limited to abstracts only, likely as a result of lack of reporting of domains in abstracts. Risk of bias in study confounding was common amongst studies due to inadequate reporting of confounding factors or matching or to accounting for confounders in analyses (Table S2).

In their assessment of the predictive power of miRNAs, some studies adjusted for potential confounders such as age, gender, and preexisting cardiovascular (CV) risk factors such as smoking status, the details of which were often unclear. Studies that considered the additional prognostic value of miRNAs also performed adjustments for hs-TnT levels, creatinine kinase (CK), N-terminal pro b-type Naturietic Peptide (NT-proBNP), and other traditional biomarkers. 

There was significant diversity amongst studies in miRNA assessed. The most common miRNAs included miR-133a, -208b, - 21, -1, -34a, -150, and -423. MiR-133, -1, and -208b have been shown to be involved in angiogenesis, smooth muscle, and cardiomyocyte differentiation [3]. MiR-21 is upregulated in cardiomyocytes and in fibroblasts. MiR-150 has been shown to be higher in patients without left ventricular remodeling [16], and MiR-423-5p is associated with congestive cardiac failure [17]. MiR-34a has been shown to be a p53-responsive miRNA [18] associated with cardiomyocyte apoptosis (Figure S1).

In terms of individual studies, 23 prospective studies reported significant differences in miRNA levels between the population, with recorded outcomes as listed above, and 16 compared the predictive power of individual or panels of miRNAs. Of these, several utilised miRNA as risk predictors of CV events or mortality post-ACS presentation. The miRNA reported in predictive modelling as biomarkers of mortality in the ACS population included miR-122-5p, -328, -134, -208b, -140-3p, -210, -132, -19b, -150, -145, -186, -499, -197, and -223. MiRNA reported as predictors of heart failure included miR-150, -101, -16, -27, -328, -134, -208b, -34a, and -652. Methodology was similar across studies in predictive modelling utilising Cox proportional hazard regression modelling, area under the receiver operating characteristic curve (ROC), and Kaplan–Meier nonparametric survival analysis. Two earlier systematic reviews assessed suggest limited clinical value to date given the diverse miRNAs investigated and endpoints reported. Cao et al. [5] analysed 12 primary research articles and found that high levels of expression of miRNAs were associated with shorter survival times. Kim et al. [6] investigated the prognostic value of miRNA signatures in CAD patients and found miR-133a to be associated with high mortality in CAD patients. Few miRNAs were studied across papers, but MiR-133a was one of the most investigated cardiomyocyte-enriched miRNAs between studies. No studies found added prognostic power of miR-133a when levels were compared to the predictive power of traditional cardiovascular risk factors or troponin levels, although Kim et al. [6] reported that miR-133a may have prognostic potential in pooled analysis. These findings are consistent with ours in demonstrating limited prognostic value of miRNA, largely due to heterogeneity of miRNA assessed and significant confounding limiting conclusive outcomes.

### 3.1. Potential Confounders

#### 3.1.1. Sampling within Studies

Although some studies took multiple samples at various time points, it was often unclear as to which samples were used for analysis. Pilbrow et al. [19] described changes in miRNA levels over time. This study was notable as it explored effects of changes in medical treatment and storage time of samples, which may result in miRNA degradation. However, a thorough investigation of these potential confounders was largely unique to this study. 

#### 3.1.2. Sample Processing Variations

As found by Boeckel, et al. [20], heparin selectively increases the degradation of certain miRNAs (miR-34a, miR-133a, miR-208, miR-378, and miR-499) within 10 minutes of administration and sustained losses (miR-34a, miR-133a, and miR-208) 1 hour post-heparinisation. This phenomenon has only been investigated in vascular and MI-related miRNA (miR-1, miR-17, miR-34a, miR-92a, miR-126, miR-133a, miR-145, miR-208, miR-378, and miR-499) in blood. Similarly, Schulte et al. [9] showed that heparin inhibits qPCR miRNA quantification, resulting in higher variation and reduced detectability of miRNA. This was shown to be reversed using heparinase for miRNA 126-3p, 223-3p, and 150-5p in a clinical AMI cohort. It was also noted that heparin suppressed Cel-miR-39, which was often used as a normalisation control which may artificially elevate expression of reported miRNAs. 

Many studies have avoided this issue by sampling blood prior to heparin administration, and some collected samples into Ethylene Diamine Tetraacetic Acid (EDTA) tubes (e.g., Grabmaier et al. [12]). However, most studies did not clearly specify how heparinisation was incorporated into their study protocol. Glinge, et al. [21] found that collection in EDTA or citrate tubes did not affect the levels of miR-1, miR-21, and miR-29b. They also found that, for sample storage at −80°C, repeat freeze–thaw cycles led to changes in miRNA levels. Although interpretation of changes in miRNA levels may not have been affected as long as a consistent methodology was applied to minimise variation, there is still uncertainty about how levels are affected by blood collection tube type or anticoagulant exposure.

#### 3.1.3. Methods of Quantifying miRNA

A major limitation of many studies stems from variations and lack of consensus for quantifying miRNA levels. Some studies utilised controls to normalise miRNA levels such as cDNA and synthetic Locked Nucleic Acid (LNA) (Cortez-Dias et al. [22]); others adjusted levels to various endogenous miRNAs [13,19,23] or utilised pooled samples [24]. Unfortunately, there are no validated internal controls such as a native miRNA level within samples, and most studies did not use a staged spike in nonhuman miRNA, a repeat sample, or a duplicate sample at two positions on the same array to avoid measurement bias. Thus, standardised quantification and dilution of samples in a meaningful manner is limited. 

Furthermore, many studies had an underlying rationale for quantifying certain miRNAs, such as to verify those found to be significantly different between healthy and patient populations in previous studies or those known to be cardiomyocyte enriched. Given that the exact mechanisms of these miRNAs are not yet elucidated, currently studied miRNAs may simply reflect existing cardiovascular prognostic markers and, thus, overlook those with independent prognostic utility. 

Few studies investigating for prognosis utilised a pilot study to determine what miRNAs could potentially be of use. Pilbrow et al. [19] was one such study, where a 375-miRNA panel was used to screen 35 ACS patients and 16 controls before those that were markedly different between the population were verified in a subsequent study of 200 patients and 100 healthy controls. In this case, downregulation of miR-652 and upregulation of miR-323-3p levels were found to add predictive power for cardiac death or heart failure to biomarkers of left ventricular ejection fraction (LVEF) and NT-proBNP. Unfortunately, no other study has subsequently investigated these miRNAs. Tang et al. [24] also performed a pilot study in 115 CAD patients to determine plasma miRNAs associated with clopidogrel antiplatelet efficacy using high-throughput illumina sequencing followed by qRT-PCR validation. These miRNAs were then assessed in a validation cohort of 1230 CAD patients to determine prediction of major adverse cardiovascular events (MACE) over a 3-year follow-up period. 

#### 3.1.4. Population-Based Confounders

Few studies disclosed detailed information about the patient population. As such, the potential for phenotypic differences complicating statistical analysis may need to be considered. There is also a lack of subgroup analyses or adjustment for differences in treatment regimes, traditional vascular risk factor status, or medical comorbidities and a lack of matched controls. However, given the complexity of post-ACS risk and the small sample sizes of these studies, an attempt to adjust for all population differences and subgroup analysis may be underpowered for detecting significant differences in miRNA expression. However, the documentation of significant between-group differences is pertinent when such differences have been previously shown to alter miRNA expression. Such an example is in the use of antiplatelet and anticoagulant therapies in ACS patients, which are known to alter miRNA expression [24,25,26]. Moreover, few studies in this review assessing post AMI prognosis commented on antiplatelet use [24] prior to blood sampling, which, in turn, could result in significant confounding in reported miRNA expression.

### 3.2. Use of a Control Population

For those studies that had a control population, healthy controls were mostly used. This is potentially problematic as population characteristics, including demographics, presence of CV risk factors, or previous history of vascular disease may drive miRNA-level differences between groups rather than the clinical presentation. With the exceptions of Alavi-Moghaddam et al., Lin et al. [13,14], and Grabmaier et al. [12], where controls were age and sex matched, and of Grabmaier et al. [12], where controls were also risk-factor matched, most studies did not have matched controls. However, most studies without a control population compared those with adverse outcomes to those without adverse outcomes within a cohort of ACS patients. This may be a better study format for prognostic assessment as this allows for direct comparison within the post-ACS population in a real-world setting where prognostication is particularly important. 

### 3.3. Prospective Studies in a Healthy Population

There are 2 prospective studies evaluating the utility of miRNAs in predicting or determining risk of ACS in a healthy population. Both studies had a follow-up period of 10 years and used a PCR panel to screen for differential miRNA expression at baseline, unlike the majority of the prospective post-AMI studies, which preselected miRNA in a biased approach.

The Nord-TrØndelag Health Study (the HUNT Study) [27,28] studied healthy individuals of which half had an MI and half did not over a 10-year follow-up period. The population was matched for cardiovascular risk factors such as their BMI, lifestyle, and socioeconomic background. In this cohort population, a panel of miR-106a-5p, miR-424-5p, let-7g-5p, miR-144-3p, and miR-660-5p added predictive value to the Framingham Risk Score (FRS) [27], and subsequent investigation found miRNA-21-5p, -26a-5p, -29c-3p, -144-3p, and -151a-5 [28] to add predictive value to the FRS. However, the use of miRNAs with other currently used stratification tools was not evaluated. The importance of using aged-matched controls is again highlighted in this cohort as miR-106a is known to negatively correlate with age [29]. Therefore, future studies should incorporate the use of age, gender, and smoking-matched controls [3]. The second prospective study in a healthy population was the Bruneck Study [30] of 820 persons. Higher miR-126 and lower miR-223 and miR-197 levels were found in those patients who had an AMI within the follow-up period. Again, this larger study did not evaluate the utility of miRNAs alongside existing tools. Moreover, the importance of confounding results with medication use is again highlighted as these miRNAs are expressed in platelets and, therefore, affected by anti-platelet agents.

### 3.4. Study Limitations

This systematic review was performed by two authors only, although conducted independently. No meta-analysis of the literature was feasible due to heterogeneity of data. Restriction of the methodology to English language and a predominant Caucasian population resulting in study selection bias was a further limitation. 

Most studies reported varied miRNA in risk prediction post-ACS. However, three studies reported miR-150 as a marker of risk prediction in ACS patients, showing prediction of CV death, left ventricular (LV) remodelling, and LV impairment [14,16,31,32]. MiR-208b was also assessed in three predictive models showing prediction of MACE over NT-proBNP and clinical syntax score in a cohort of AMI patients over a 3-year follow-up [33] and was superior to NT-proBNP in predicting LV remodelling [34] as well as in predicting mortality post AMI at 6 months [13]. MiR-208b has been shown to be upregulated in AMI patients, released immediately post PCI, and produced in rat myocardium as a marker of myocardial injury [13] and is upregulated in the myocardium of dilated cardiomyopathy patients [35], supporting its expression as representative of human cardiomyocyte growth/death. 

It is important to note that most studies did not utilise a validated risk scoring system or a comprehensive panel of cardiovascular risk factors. For example, isolated findings lost significance after adjusting for common cardiovascular risk factors (e.g., Widera, et al. [36] and Eitel, et al. [37]). In contrast, some risk prediction models of outcome based on clinical variables and traditional biomarkers were improved with miRNA, such as miR-208b and -34a [34] and miR-150 [16] in LV function and size. These data point to a role of certain miRNA to improve risk prediction alongside traditional biomarkers and clinical risk predictors in ACS patients.

### 3.5. Future Studies

Future studies looking at miRNA as prognostic markers in ACS should be a minimum of 12 months in duration and ideally longer follow-up to adequately assess CV morbidity such as stent restenosis, bleeding, ischaemic events, and heart failure as well as mortality with combined and well-defined endpoints [38]. They should have an unbiased approach to miRNA selection and stringent quality checks during sample processing and miRNA measurements, such as staged nonhuman spike in miRNA and duplicate and repeat sampling on arrays to avoid measurement bias [39]. They should account for known confounders including CV risk factors and medications. Moreover, they should have low level of bias and adequate sample size to detect differences in a heterogenous population. Another important consideration is ensuring reproducibility in data by using discovery and validation cohorts. Such an approach will help in identifying significantly dysregulated microRNAs in a discovery cohort followed by confirmation of these findings in a separate, bigger validation cohort.

Few studies assessed in this review came close to achieving these goals. Several studies had reasonable follow-up times of at least 12 months [12,14,15,17,19,22,24,33,34,40,41,42,43,44,45,46,47,48], and of those, most had reasonable sample size except for Liu et al. [44], Jantti et al. [43], Matsumoto et al. [46], and Costa et al. [40]. Low level of bias was reported across all domains in five of these studies with adequate follow-up and reasonable sample size, including studies by Lv et al., Pilbrow et al., Schulte et al., Mayer et al., and Tang et al. [15,19,24,34,48]. Of these 5 studies, all but 2 utilised previous literature search for miRNA selection. Given this relatively new field with a lack of knowledge of biological mechanisms of miRNA to date, this is limiting in its interpretation of miRNA as prognostic markers in ACS, although not taking away from the studies on which these selections were based [30,49,50,51,52]. It is important in this developing field to utilise an unbiased approach to investigation of miRNA as undertaken by Tang et al. and Pilbrow et al. Pilbrow et al. did not assess MACE but did assess readmission for heart failure and mortality as prognostic outcomes in ACS, a limitation in this study. Tang et al. utilised a discovery and validation approach and assessed MACE in ACS patients. However, this was only assessed for 6 candidate miRNAs associated with clopidogrel antiplatelet efficacy and, thus, quite a specific group of miRNAs for prediction of MACE in an ACS population. 

## 4. Conclusions

Taken together, our findings do not support the use of miRNA for prognostication post-ACS beyond traditional cardiovascular risk factors, existing risk scores, and stratifications tools currently. The main barriers include small sample sizes, bias selection of miRNAs already known to be implicated in cardiovascular disease, and inadequate efforts to ensure data reproducibility. Future studies will need to be considerably larger with longer follow-up times beyond 12 months, to incorporate the efficient use of screening panels to detect differences in miRNAs of unknown significance, and to allow for a more comprehensive assessment of potential confounders and well-defined outcomes including ischaemia, restenosis, bleeding events, and heart failure alongside mortality. Moreover, greater consistency in methodology, reporting of population selection, patient characteristics, and statistical consideration of comorbid factors and potential confounders is required to clearly determine whether miRNAs have prognostic utility in this setting. 

## Supplementary Materials

The following are available online at https://www.mdpi.com/2073-4409/8/12/1572/s1, Figure S1: Number of studies investigating each miRNA, Table S1: Study characteristics, Table S2: QUIPS risk of bias in study assessment summary table.
