# Supplementary material for: MicroRNAs as Prognostic Markers in Acute Coronary Syndrome Patients—A Systematic Review
Source: Cells. 2019 Dec 4;8(12):1572. doi: 10.3390/cells8121572 (PMC6952952; doi:10.3390/cells8121572)
Supplement: Supplementary file 1 [file cells-08-01572-s001.zip › Table S1.docx]

| Study | Population | Control population | Total sample size | Relevant outcome measures | miRNA | Comparator | Follow up time | Retrospective v Prospective | Y/N predictor of cardiac death |
| --- | --- | --- | --- | --- | --- | --- | --- | --- | --- |
| Widera, Gupta et al. (2011) | Patients admitted with ACS (hs-cTnT and clinical picture) | - | 444 | All cause mortality | MiR-1, miR-133a, miR-133b, miR-208a, miR-208b, and miR-499 | hs-cTnT | 6 months | P | n/a – no added prognostic information |
| Eitel, Adams et al. (2012) | STEMI admission, receiving angioplasty | - | 216 | MACE - death, reinfarction, and new congestive heart failure | miR-133a | Cardiac magnetic resonance (CMR) imaging and clinical characteristics | 6 months | P | n/a – no added prognostic information |
| Costa, Cortez-Dias et al. (2012) | AMI patients from OACIS registry between 1998 and 2009 | - | 42 | Death, re-infarction or hospitalization for cardiac causes | miR-375* nil comparator | Nil | 1-2 years | P | Yes – high levels |
| Matsumoto, Sakata et al. (2012) | 19 in cardiac death group + 21 survival group | - | 40 | Cardiac death | miR-155 and miR-380* | Nil | 3 years | R | [miR- 155] and [miR-380⁄*] were approximately 4- and 3-fold higher in population with cardiac death within 1yr post discharge |
| Devaux, Vausort et al. (2013) | Patients admitted with AMI | - | 150 | LV contractility | miR-16, miR-27a, miR-101 and miR-150 | Multi-parameter clinical model + NtproBNP | 6 months | P | Yes - added to the predictive value of their model for outcome  Upregulated miR-16, miR-27a  Downregulated miR-101, miR-150 |
| Bauters, Kumarswamy et al. (2013) | First presentation AMI + consistent medical treatment regime | - | 246 | Correlation with cardiac biomarkers + LV remodelling | miR-133a and miR-423-5p | cardiac biomarkers (BNP, CRP, and cardiac troponin I) | 1 year | P | n/a - association |
| Gidlof, Smith et al. (2013) | Suspected ACS presenting to ED | - | 407 | Mortality, heart failure, LVEF | miR-1, miR-208b and miR-499-5p | TnT | 30 days | P | n/a - association |
| Goretti, Vausort et al. (2013) | First presentation of STEMI | - | 30 | LV function and remodeling after MI (change in LV end-diastolic volume (ΔEDV)) | miR-150 alongside NT-proBNP | NT-proBNP alone | 4 months | P | Yes downregulated miR-150 |
| Devaux, Vausort et al. (2013) | 21 AMI patients who developed HF | 65 AMI patients with no HF at 1 yr | 86 | Heart failure | miR-192, miR-194, and miR-34a | Nil | 1 year | R | Yes upregulated miR-192, miR-194, and miR-34a |
| Matsumoto, Sakata et al. (2013) | 359 AMI patients | 30 healthy controls | 389 | Composite of cardiogenic death + heart failure | miR-328, miR-134 | hs-cTnT | 6 months | P | Yes – upregulated miR-328, miR-134.  Greater sensitivity and specificity |
| He, Lv et al. (2014) | 359 First presentation AMI | - | 359 | Mortality, heart failure | miR-208b and miR-34a | NT-proBNP as comparator | 6 months | P | Yes – upregulated miR-208b and miR-34a |
| Lv, Zhou et al. (2014) | 200 patients admitted with STEMI | 100 healthy controls | 300 | Cardiac death, heart failure | miR-323-3p, miR-652, miR-27b, miR-103 and miR-208a | biomarkers of LVEF and NT-proBNP | 5 years | P | Yes – miR-652 downregulated, miR-323-3p levels remained elevated +1yr stable biomarker for ACS |
| Pilbrow, Cordeddu et al. (2014) | 142 NSTEMI patients | - | 142 | 1yr and 2yr cardiovascular mortality | miR-499-5p and miR-21 | Single point hs-cTnT | 2 years | P | Yes – upregulated miR-499-5p,  No for miR-21 |
| Olivieri, Antonicelli et al. (2014) | 246 first STEMI, successful PCI; life expectancy >1yr | - | 246 | MACE - heart failure and cardiovascular hospitalisation/death | miR-145 | NT-proBNP, CK-MB individually | 1 year | P | Yes – upregulated miR-145 |
| Dong, Liu et al. (2015) | 873 Documented CAD patients (diagnostic angiography) | - | 873 | Cardiovascular death | miR-126, miRNA-197 and miRNA-223 | conventional cardiovascular risk factors (BMI, diabetes mellitus, HTN, history of MI, hyperlipidemia, ever smoker) | 4 years (median) | P | Yes – miR-197, miR-223 for ACS group (not for SAP group) |
| Schulte, Molz et al. (2015) | 1155 Patients presenting with acute chest pain to ED | - | 1155 | All cause mortality or MI | miR-133a, miR-208b, miR-223, miR-320a, miR-451 and miR-499 | Nil | 2 years | P | No – borderline significance for high miR-208b for 30 day mortality |
| Devaux, Mueller et al. (2015) | 142 STEMI treated with angioplasty  single-centre | 18 healthy controls | 160 | Adverse cardiovascular events – death, MI, unstable angina, stroke or hospitalization due to acute heart failure. | Serum miR-1-3p, -122-5p, -133a-3p, -133b, -208b-3p and -499a-5p at the time of cardiac catheterization | CKMB, NT-proBNP, renal (Cr, urea, uric acid and eGFR), liver biomarkers (ALT, AST, γ-GT, bilirubin), inflammatory (WBC and neutrophil counts, CRP, IL-6) and HbA1C. Troponin I | 20.8 months | P | Yes – higher Circulating miR-122-5p/133b ratio |
| Cortez-Dias, Costa et al. (2016) | 316 patients | 67 healthy controls | 383 | MI, hospitalisation for unstable angina, stroke, revascularization procedures or heart failure requiring hospitalisation. | miR-133a | Age, gender, hypertension diabetes, and smoking adjustment  Nil comparator | 2 years | P | Lower cumulative survival for high levels of miR-133a |
| Ke-Gang and Xue-Jing (2016) | 179 cardiogenic shock patients | - | 179 | All cause mortality | miR21, miR122a, miR320a and miR423 | Nil | 90 days | P | Yes – higher levels |
| Jantti, Segersvard et al. (2016) | 111 patients undergoing coronary angiography (82 with stable CAD and 29 with ACS) | - | 111 | MACE: death, nonfatal myocardial infarction, and need for revascularisation | miR133a | Nil | 32 months (median) | P | No added prognostic information |
| De Rosa, De Rosa et al. (2017) | 44 patients (SITAGRAMI trial) | 18 matched controls | 62 | Adverse ventricular remodelling (AVR) + combined cardiovascular endpoint (cardiovascular death, MI, stroke, CABG, coronary re-intervention, rehospitalisation for UA) | miR-1, miR-21, miR-29b and miR-92a | MRI parameters for AVR - infarct volume, LVEF, LVEDV | 6 months | P | Nil clinical outcome prediction |
| Grabmaier, Clauss et al. (2017) | 1112 patients (Atherogene study) 430 ACS and 682 SA | - | 1112 | Mortality | miR-19a, miR-19b, miR-132, miR-140-3p, miR-142-5p, miR-150, miR-186, and miR-210 | Troponin, NT-proBNP, LVEF, and numbers of affected vessels. | 4 years | P | Yes – for mortality in ACS (Except miR-142-5p) |
| Karakas, Schulte et al. (2017) | 100 AMI and 80 UA | 80 healthy controls | 260 | Left ventricular remodelling, MACE | miR-208b | Nil | 6 months | P | Yes - upregulated |
| Liu, Yuan et al. (2017) | 72AMI + 10 coronary disease | 10 healthy control | 92 | MACE | miR-184 | Nil | 1 year | P | Yes- upregulated |
| Liu, Sun et al. (2017) | 104 CAD | 50 healthy controls | 154 | Mortality, cardiovascular events | miR-23a | Nil | 1 year | P | Yes - upregulated |
| Toni Antti Juhani Jantti, Segersvard et al. (2017) | 179 patients (European multi-center CardShock study) | - | 179 | Mortality | miR-423-5p | CardShock risk stratification + hsTnT, ALT, NTproBNP, and lactate), clinical data | 90 days | P | Yes - upregulated |
| Zhang, Lang et al. (2017) | 140 AMI patients who had PCI | - | 140 | MACE - death, HF, cardiogenic shock/ hypotension, malignant arrhythmia, recurrent myocardial infarction, and LVEF<50%. | miR-208b-3p | hsCRP, NT-proBNP, Clinic SYNTAX score (CSS - calculated by coronary angiograph and clinical data), LVEF (Echo) | 3 years | P | Yes - upregulated |
| Alavi-Moghaddam, Chehrazi et al. (2018) | 21 STEMI patients | 8 age and gender matched controls | 30 | Survival post AMI at 6 months | miR-208b | Cardiovascular risk factors smoking, DM, HTN, Hyperlipidaemia, Reduced EF, age, gender, cTnT | 6 months | P | Yes – upregulated  MiR-208b predicting death at 6 months post AMI. |
| Liu, Niu et al. (2018) | 145 NSTEMI | 30 control (non matched) | 175 | Angina, reinfarction, arrhythmia | miR-1, 133, 208, 499 | cTnT | 10days | P | Yes miR-499 upregulation |
| Lin, Zhang et al. (2019) | 113 (54 Post AMI CCF, 59 post AMI no CCF) | 59 healthy controls (age and sex matched) | 172 | Post AMI CCF | miR-29a, miR-133a, miR-208b, miR-499, miR-150, miR-194, miR-  192, and miR-34a | BNP | 1 year | R | Yes miR-150 down regulation in post MI CCF;  miR-150 added to prognosis alongside biomarker BNP in predicting CCF post AMI at 1 year |
| Mayer, Seidlerova et al. (2019) | 826 (487 CAD and 339 CVA patients) |  | Pilot study 100 patients  Validation study 5miRNA’s - | All cause mortality  CVS mortality | miR-133a, 1, 21, 34a, 126, mmu-miR 499, miR-223, 197, 19a, 214 (pilot)  miR-1,19,126,133, 223 (validation cohort) | BNP, cTnI, | 6-36 months after ACS/Revasc/CVA) interview - Follow up mean 2050 days (5.6yrs) | P | Yes miR-1, -19, -126, -133, -223 associated with 5yr all cause death and CVS mortality.  miR-19a downregulation predicted CVS and all cause mortality in CAD patients at 5 years when accounting for other miRNA |
| Tang, Lei et al. (2019) | 115 CAD patients pilot and 1230 CAD patients validation | nil | 1199 | MACE – CVS death, MI, stent thrombosis  Bleeding events on dual antiplatelets in CAD post PCI | miR-126, 130a, 142, 27a, 21, 106a | CVS RF, Medications – CCB’s,BB’s, ACE-I, PPI’s, biochemical parameters including CK, Lipids. | 3 years | P | miR-142 upregulated in MACE  miR-126-5p upregulated in MACE |

**Table S1. Study Characteristics**

**References:**

Alavi-Moghaddam, M., M. Chehrazi, S. D. Alipoor, M. Mohammadi, A. Baratloo, M. P. Mahjoub, M. Movasaghi, J. Garssen, I. M. Adcock and E. Mortaz (2018). "A Preliminary Study of microRNA-208b after Acute Myocardial Infarction: Impact on 6-Month Survival." Disease Markers **2018**: 2410451.

Bauters, C., R. Kumarswamy, A. Holzmann, J. Bretthauer, S. D. Anker, F. Pinet and T. Thum (2013). "Circulating miR-133a and miR-423-5p fail as biomarkers for left ventricular remodeling after myocardial infarction." International Journal of Cardiology **168**(3): 1837-1840.

Cortez-Dias, N., M. C. Costa, P. Carrilho-Ferreira, D. Silva, C. Jorge, C. Calisto, T. Pessoa, S. R. Martins, J. C. de Sousa, P. C. da Silva, M. Fiuza, A. N. Diogo, F. J. Pinto and F. J. Enguita (2016). "Circulating miR-122-5p/miR-133B ratio is a specific early prognostic biomarker in acute myocardial infarction." Circulation Journal **80**(10): 2183-2191.

Costa, M., N. Cortez-Dias, P. Carrilho-Ferreira, D. Silva, C. Jorge, R. Placido, C. Calisto, M. Fiuza, A. Nunes Diogo and F. J. Enguita (2012). "miR-375: Novel biomarker for early prognostic stratification of acute myocardial infarction." Cardiovascular Research **93**: S11.

De Rosa, R., S. De Rosa, D. Leistner, J. N. Boeckel, T. Keller, S. Fichtlscherer, S. Dimmeler and A. M. Zeiher (2017). "Transcoronary Concentration Gradient of microRNA-133a and Outcome in Patients With Coronary Artery Disease." American Journal of Cardiology **120**(1): 15-24.

Devaux, Y., M. Mueller, P. Haaf, E. Goretti, R. Twerenbold, J. Zangrando, M. Vausort, T. Reichlin, K. Wildi, B. Moehring, D. R. Wagner and C. Mueller (2015). "Diagnostic and prognostic value of circulating microRNAs in patients with acute chest pain." Journal of Internal Medicine **277**(2): 260-271.

Devaux, Y., M. Vausort, G. P. McCann, D. Kelly, O. Collignon, L. L. Ng, D. R. Wagner and I. B. Squire (2013). "A panel of 4 microRNAs facilitates the prediction of left ventricular contractility after acute myocardial infarction.[Erratum appears in PLoS One. 2013;8(8). doi:10.1371/annotation/458a1f6a-6327-429a-81cb-992c97f04bd6]." PLoS ONE [Electronic Resource] **8**(8): e70644.

Devaux, Y., M. Vausort, G. P. McCann, J. Zangrando, D. Kelly, N. Razvi, L. Zhang, L. L. Ng, D. R. Wagner and I. B. Squire (2013). "MicroRNA-150: A novel marker of left ventricular remodeling after acute myocardial infarction." Circulation: Cardiovascular Genetics **6**(3): 290-298.

Dong, Y. M., X. X. Liu, G. Q. Wei, Y. N. Da, L. Cha and C. S. Ma (2015). "Prediction of long-term outcome after acute myocardial infarction using circulating miR-145." Scandinavian Journal of Clinical & Laboratory Investigation **75**(1): 85-91.

Eitel, I., V. Adams, P. Dieterich, G. Fuernau, S. de Waha, S. Desch, G. Schuler and H. Thiele (2012). "Relation of circulating MicroRNA-133a concentrations with myocardial damage and clinical prognosis in ST-elevation myocardial infarction." American Heart Journal **164**(5): 706-714.

Gidlof, O., J. G. Smith, K. Miyazu, P. Gilje, A. Spencer, S. Blomquist and D. Erlinge (2013). "Circulating cardio-enriched microRNAs are associated with long-term prognosis following myocardial infarction." BMC Cardiovascular Disorders **13**: 12.

Goretti, E., M. Vausort, D. R. Wagner and Y. Devaux (2013). "Association between circulating microRNAs, cardiovascular risk factors and outcome in patients with acute myocardial infarction." Int J Cardiol **168**(4): 4548-4550.

Grabmaier, U., S. Clauss, L. Gross, I. Klier, W. M. Franz, G. Steinbeck, R. Wakili, H. D. Theiss and C. Brenner (2017). "Diagnostic and prognostic value of miR-1 and miR-29b on adverse ventricular remodeling after acute myocardial infarction - The SITAGRAMI-miR analysis." International Journal of Cardiology **244**: 30-36.

He, F., P. Lv, X. Zhao, X. Wang, X. Ma, W. Meng, X. Meng and S. Dong (2014). "Predictive value of circulating miR-328 and miR-134 for acute myocardial infarction." Molecular & Cellular Biochemistry **394**(1-2): 137-144.

Jantti, T. A. J., H. Segersvard, J. P. Lassus, Y. Devaux, M. Vausort, K. Immonen, K. Pulkki, I. Tikkanen, P. Lakkisto and V. P. Harjola (2016). "Circulating microRNAs miR-21, miR-122a, miR-320a and miR-423 predict mortality in patients with cardiogenic shock." European Heart Journal **37 (Supplement 1)**: 321.

Karakas, M., C. Schulte, S. Appelbaum, F. Ojeda, K. J. Lackner, T. Munzel, R. B. Schnabel, S. Blankenberg and T. Zeller (2017). "Circulating microRNAs strongly predict cardiovascular death in patients with coronary artery disease-results from the large AtheroGene study." European Heart Journal **38**(7): 516-523.

Ke-Gang, J., Zhi-Wei, L, Xin, Z, Jing, W, Ping, S, and H. Xue-Jing, Hong-Xia, T, Xin, T, and Xiao-Cheng, L (2016). "Evaluating Diagnostic and Prognostic Value of Plasma miRNA133a in Acute Chest Pain Patients Undergoing Coronary Angiography.".

Lin, X., S. Zhang and Z. Huo (2019). "Serum circulating miR-150 is a predictor of post-acute myocardial infarction heart failure." International Heart Journal **60**(2): 280-286.

Liu, G., X. Niu, X. Meng and Z. Zhang (2018). "Sensitive miRNA markers for the detection and management of NSTEMI acute myocardial infarction patients." Journal of Thoracic Disease **10**(6): 3206-3215.

Liu, X., L. Yuan, F. Chen, L. Zhang, X. Chen, C. Yang and Z. Han (2017). "Circulating miR-208b: A Potentially Sensitive and Reliable Biomarker for the Diagnosis and Prognosis of Acute Myocardial Infarction." Clinical Laboratory **63**(1): 101-109.

Liu, Z. H., X. P. Sun, S. L. Zhou and H. X. Wang (2017). "Research on the relations between the variation of miRNA-184 before and after treatment of acute myocardial infarction and prognosis." European Review for Medical & Pharmacological Sciences **21**(4): 843-847.

Lv, P., M. Zhou, J. He, W. Meng, X. Ma, S. Dong, X. Meng, X. Zhao, X. Wang and F. He (2014). "Circulating miR-208b and miR-34a are associated with left ventricular remodeling after acute myocardial infarction." International Journal of Molecular Sciences **15**(4): 5774-5788.

Matsumoto, S., Y. Sakata, D. Nakatani, S. Suna, H. Mizuno, M. Shimizu, M. Usami, T. Sasaki, H. Sato, Y. Kawahara, T. Hamasaki, S. Nanto, M. Hori and I. Komuro (2012). "A subset of circulating microRNAs are predictive for cardiac death after discharge for acute myocardial infarction." Biochemical & Biophysical Research Communications **427**(2): 280-284.

Matsumoto, S., Y. Sakata, S. Suna, D. Nakatani, M. Usami, M. Hara, T. Kitamura, T. Hamasaki, S. Nanto, Y. Kawahara and I. Komuro (2013). "Circulating p53-responsive microRNAs are predictive indicators of heart failure after acute myocardial infarction." Circulation Research **113**(3): 322-326.

Mayer, O., Jr., J. Seidlerova, V. Cerna, A. Kucerova, J. Vanek, P. Karnosova, J. Bruthans, P. Wohlfahrt, R. Cifkova, M. Pesta and J. Filipovsky (2019). "The low expression of circulating microRNA-19a represents an additional mortality risk in stable patients with vascular disease." International Journal of Cardiology **289**: 101-106.

Olivieri, F., R. Antonicelli, L. Spazzafumo, G. Santini, M. R. Rippo, R. Galeazzi, S. Giovagnetti, Y. D'Alessandra, F. Marcheselli, M. C. Capogrossi and A. D. Procopio (2014). "Admission levels of circulating miR-499-5p and risk of death in elderly patients after acute non-ST elevation myocardial infarction." International Journal of Cardiology **172**(2): e276-e278.

Pilbrow, A. P., L. Cordeddu, V. A. Cameron, C. M. Frampton, R. W. Troughton, R. N. Doughty, G. A. Whalley, C. J. Ellis, T. G. Yandle, A. M. Richards and R. S. Y. Foo (2014). "Circulating miR-323-3p and miR-652: Candidate markers for the presence and progression of acute coronary syndromes." International Journal of Cardiology **176**(2): 375-385.

Schulte, C., S. Molz, S. Appelbaum, M. Karakas, F. Ojeda, D. M. Lau, T. Hartmann, K. J. Lackner, D. Westermann, R. B. Schnabel, S. Blankenberg and T. Zeller (2015). "miRNA-197 and miRNA-223 Predict Cardiovascular Death in a Cohort of Patients with Symptomatic Coronary Artery Disease." PLoS ONE [Electronic Resource] **10**(12): e0145930.

Tang, Q. J., H. P. Lei, H. Wu, J. Y. Chen, C. Y. Deng, W. S. Sheng, Y. H. Fu, X. H. Li, Y. B. Lin, Y. L. Han and S. L. Zhong (2019). "Plasma miR-142 predicts major adverse cardiovascular events as an intermediate biomarker of dual antiplatelet therapy." Acta Pharmacologica Sinica **40**(2): 208-215.

Toni Antti Juhani Jantti, T. A. J., H. Segersvard, T. Tarvasmaki, J. Lassus, Y. Devaux, M. Vausort, K. Immonen, K. Pulkki, I. Tikkanen, P. Lakkisto and V. P. Harjola (2017). "MicroRNA-423-5p levels correlate with markers of hypoperfusion and organ injury and are associated with 90-day mortality in cardiogenic shock." European Journal of Heart Failure **19**: 589.

Widera, C., S. K. Gupta, J. M. Lorenzen, C. Bang, J. Bauersachs, K. Bethmann, T. Kempf, K. C. Wollert and T. Thum (2011). "Diagnostic and prognostic impact of six circulating microRNAs in acute coronary syndrome." Journal of Molecular & Cellular Cardiology **51**(5): 872-875.

Zhang, Y., M. Lang and L. Xie (2017). "The role of plasma miR-208b-3p in the prognostic and risk stratification of acute myocardial infarction." Journal of the American College of Cardiology **70 (16 Supplement 1)**: C92.
