# Supplementary material for: MicroRNAs as Prognostic Markers in Acute Coronary Syndrome Patients—A Systematic Review
Source: Cells. 2019 Dec 4;8(12):1572. doi: 10.3390/cells8121572 (PMC6952952; doi:10.3390/cells8121572)
Supplement: Supplementary file 1 [file cells-08-01572-s001.zip › Table S2.docx]

**Table S2. QUIPS Risk of Bias in Studies Assessment Summary Table**

| **Study** | **Study participation** | **Study attrition** | **Prognostic factor measurement** | **Outcome measurement** | **Study confounding** | **Statistical analysis and reporting** |
| --- | --- | --- | --- | --- | --- | --- |
| **Widera, C et al. (2011)** | Low | Low | Low | Low | Moderate | Low |
| **Eitel, I et al. (2012)** | Low | Low | Low | Low | Low | Low |
| **Costa, M et al. (2012)** | Moderate | High | Low | Moderate | High | Moderate |
| **Matsumoto, S et al. (2012)** | Low | High | Low | Moderate | Low | Low |
| **Devaux, Y et al. (2013) (a)** | Low | Low | Low | Low | Low | Low |
| **Bauters, C et al. (2013)** | Low | Moderate | Low | Low | Low | Low |
| **Gidlof, O et al. (2013)** | Moderate | Moderate | Low | Low | Low | Low |
| **Goretti, E et al. (2013)** | Low | Low | Moderate | Low | Low | Moderate |
| **Devaux, Y et al. (2013) (b)** | Low | Low | Low | Low | Low | Low |
| **Matsumoto, S et al. (2013)** | Low | Low | Low | Low | Moderate | Low |
| **He, F et al. (2014)** | Low | Low | Low | Moderate | Low | Low |
| **Lv, P et al. (2014)** | Low | Low | Low | Low | Low | Low |
| **Pilbrow, A.P et al. (2014)** | Low | Low | Low | Low | Low | Low |
| **Olivieri, F et al. (2014)** | Low | Moderate | Low | Moderate | Low | Moderate |
| **Dong, Y.M et al. (2015)** | Moderate | Moderate | Low | Low | Low | Low |
| **Schulte, C et al. (2015)** | Low | Low | Low | Low | Low | Low |
| **Devaux, Y et al. (2015)** | Low | Moderate | Low | Low | Moderate | Low |
| **Cortez-Dias, N et al. (2016)** | Low | Low | Low | Low | Moderate | Low |
| **Ke-Gang, J et al (2016)** | Low | Low | Low | Low | Low | Low |
| **Jantti, T.AJ et al. (2016)** | Moderate | Low | High | Low | Moderate | Low |
| **De Rosa, R et al. (2017)** | Moderate | Low | Low | Low | Low | Low |
| **Grabmaier, U et al. (2017)** | Low | Low | Low | Low | Moderate | Low |
| **Karakas, M et al. (2017)** | Low | Low | Low | Low | Low | Low |
| **Liu, X et al. (2017)** | High | High | Low | Moderate | High | High |
| **Liu, Z.H et al. (2017)** | Moderate | High | Low | Low | High | High |
| **Toni Antti Juhani Jantti, T.AJ et al. (2017)** | High | Low | Low | Low | Moderate | Low |
| **Zhang, Y et al. (2017)** | High | Low | Low | Low | Moderate | Moderate |
| **Alavi-Moghaddam, M et al. (2018)** | Low | Low | Low | Low | Moderate | Low |
| **Liu, G et al. (2018)** | Low | Low | Low | Moderate | High | High |
| **Lin, X et al. (2019)** | Moderate | Low | Low | Low | Low | Low |
| **Mayer, O et al. (2019)** | Low | Low | Low | Low | Low | Low |
| **Tang, Q.J et al. (2019)** | Low | Low | Low | Low | Low | Low |
