# Supplementary figures and images for: MicroRNAs as Prognostic Markers in Acute Coronary Syndrome Patients—A Systematic Review
Source: Cells. 2019 Dec 4;8(12):1572. doi: 10.3390/cells8121572 (PMC6952952; doi:10.3390/cells8121572)

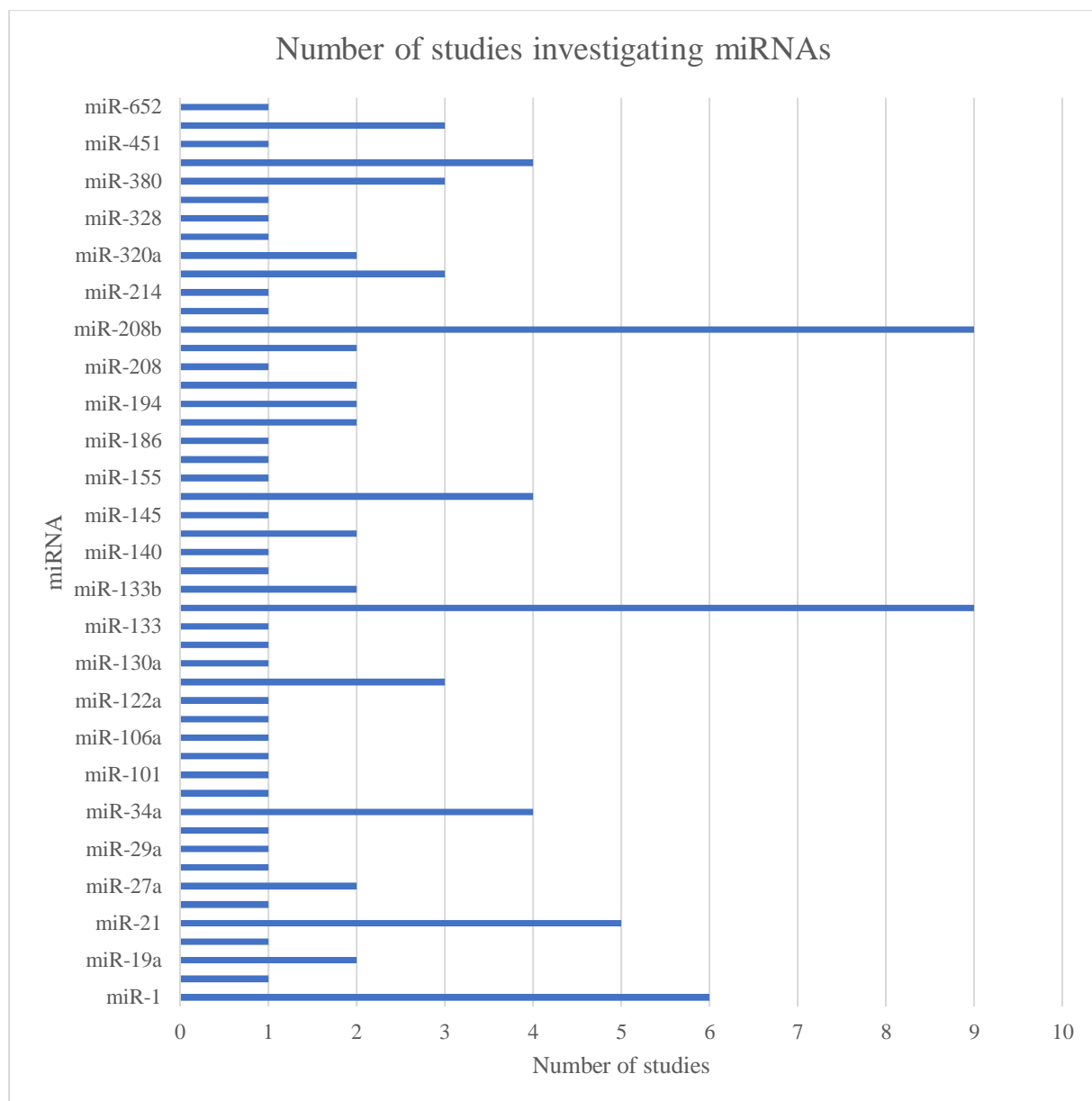

Supplement: Supplementary file 1 [file cells-08-01572-s001.zip › FigureS1.pdf]
